# *In Vitro* Interactions between Non-Steroidal Anti-Inflammatory Drugs and Antifungal Agents against Planktonic and Biofilm Forms of *Trichosporon asahii*

**DOI:** 10.1371/journal.pone.0157047

**Published:** 2016-06-08

**Authors:** Suteng Yang, Yong Liao, Lin Cong, Xuelian Lu, Rongya Yang

**Affiliations:** 1 Department of Dermatology, General Hospital of Beijing Military Command, Beijing, China; 2 The Clinical Medical College in the Beijing Military Region, Second Military Medical University of People’s Liberation Army, Shanghai, China; University at Buffalo, SUNY, UNITED STATES

## Abstract

Increasing drug resistance has brought enormous challenges to the management of *Trichosporon spp*. infections. The *in vitro* antifungal activities of non-steroidal anti-inflammatory drugs (NSAIDs) against *Candida spp*. and *Cryptococcus spp*. were recently discovered. In the present study, the *in vitro* interactions between three NSAIDs (aspirin, ibuprofen and diclofenac sodium) and commonly used antifungal agents (fluconazole, itraconazole, voriconazole, caspofungin and amphotericin B) against planktonic and biofilm cells of *T*. *asahii* were evaluated using the checkerboard microdilution method. The spectrophotometric method and the XTT reduction assay were used to generate data on biofilm cells. The fractional inhibitory concentration index (FICI) and the *ΔE* model were compared to interpret drug interactions. Using the FICI, the highest percentages of synergistic effects against planktonic cells (86.67%) and biofilm cells (73.33%) were found for amphotericin B/ibuprofen, and caspofungin/ibuprofen showed appreciable percentages (73.33% for planktonic form and 60.00% for biofilm) as well. We did not observe antagonism. The *ΔE* model gave consistent results with FICI (86.67%). Our findings suggest that amphotericin B/ibuprofen and caspofungin/ibuprofen combinations have potential effects against *T*. *asahii*. Further *in vivo* and animal studies to investigate associated mechanisms need to be conducted.

## Introduction

*Trichosporon spp*. are basidiomycetous yeast-like anamorphic organisms that are widely distributed in nature and can be found mainly in tropical and temperate regions [1]. Most *Trichosporon spp*. routinely isolated in clinical laboratories are related to episodes of colonization or superficial infections; however, these fungi have been recognized as emergent opportunistic pathogens that cause invasive infections worldwide, mainly in immunocompromised hosts [2]. Invasive trichosporonosis is an uncommon but frequently fatal fungal infection in immunocompromised patients, particularly in those with hematological malignancies, and *Trichosporon asahii* (*T*. *asahii*) is the main pathogen [1,3]. Despite the increasing relevance of the genus *Trichosporon* in contemporary medicine, treating patients with invasive trichosporonosis remains a challenge. Previous studies have found that *T*. *asahii* initially resistant to caspofungin (CAS), more resistant to amphotericin B (AMB), and more sensitive to azoles than other *Trichosporon* species [4,5]. Though azoles, especially voriconazole (VOR), demonstrate *in vitro* and *in vivo* effects on clinical isolates of *T*. *asahii*, *in vitro* azole-resistant and even pan-resistant isolates have already been discovered [6,7], and treatment failure with fluconazole (FLU) has been reported [6–8]. In addition, some invasive infections with *Trichosporon* spp. are typically associated with invasive medical devices, especially central venous catheters [9,10]. The ability of *T*. *asahii* to adhere and form biofilms that are structured microbial communities embedded in an extracellular polymeric substance (EPS) on implanted devices is an important possible reason why the strains have markedly enhanced resistance to antifungal agents and avoid host immune responses [9]. Although azoles have been effective against *T*. *asahii* planktonic cells, they have failed to eradicate the preformed biofilms [9], which may bring about treatment failure. However, despite an insistent demand, the development of new antifungal agents for clinical therapy has lagged behind the increasing incidence of drug resistance [11].

Antifungal combined therapy can achieve broader antifungal coverage and potentially reduce acquired resistance; nevertheless, it should be noted that the azoles and polyenes combination haved been reported to have an antagonistic effect *in vitro* [12]. The use of non-antifungal agents such as non-steroidal anti-inflammatory drugs (NSAIDs), antimicrobials, calcium homeostasis regulators and other agents combined with fluconazole against planktonic cells as well as biofilms of *C*. *albicans* has been reported [11]. NSAIDs, including aspirin (ASA), diclofenac sodium (DIC), and ibuprofen (IBR), are commonly used to ameliorate fever and other symptoms of illness. Activities of ASA against *C*. *albicans* biofilms and *Cryptococcus spp*. planktonic cells have been observed [13,14]. The results from these studies indicated that the NSAIDs have potential antifungal activities against pathogenic fungi; these potential antifungal activities are believed to be associated with changes in prostaglandin production, membrane potentials, biofilm formation [1,9] and reduction of extracellular polysaccharide [14], which may provide clues for a combination strategy against *T*. *asahii*. No study has so far focused on the interactions between NSAIDs and antifungal agents against planktonic and biofilm cells of *T*. *asahii*. The objective of this study was to measure the *in vitro* efficacy of NSAIDs alone and in combination with antifungal agents against *T*. *asahii*, which can help us find the appropriate combinations against *T*. *asahii* planktonic and biofilms cells.

## Materials and Methods

### Species and culture conditions

CBS 2479 (*T*. *asahii* type strain) was obtained from the CBS-KNAW Fungal Biodiversity Centre (the Netherlands), and *T*. *asahii* clinical strains (701, 702, 703, 704, 901, 902, 06198 and 06674) were isolated from patients with trichosporonosis. The identification of isolates was performed using the commercial system (API 20C AUX, BioMérieux, France) and DNA sequencing of the intergenic spacer 1 (IGS1, GenBank: AB066386.1) region of the rRNA gene. Six *T*. *asahii* isolates with high FLU MIC values (HFM-isolates) were induced as previously reported by culturing CBS 2479 in medium containing fluconazole at concentrations from 4.0 to 16 μg/ml [15]. After 48 h of culture on Sabouraud’s Agar (SDA, Sigma, Shanghai, China) at 35°C, the strains were cultured aerobically at 35°C for 24 h on Yeast Extract Peptone Dextrose (YPD, Sigma, Shanghai, China) on an orbital shaker (130 rpm). The cells were harvested, washed three times with phosphate-buffered saline (PBS pH 7.2), re-suspended in RPMI 1640 (Sigma, Shanghai, China) that had been adjusted to pH 7.0 with 0.165 M morpholinepropanesulfonic acid (MOPS, Sigma, Shanghai, China) to densities of 10^3^ CFU/ml for the planktonic cell study and 10^6^ CFU/ml for the biofilm study and counted with a hemocytometer.

### Drug solutions

The stock solutions of FLU (Sigma, 100 mg/ml), itraconazole (ITC, Sigma, 100 mg/ml), VOR (Sigma, 100 mg/ml), CAS (Sigma, 100 mg/ml), AMB (Sigma, 100 mg/ml), ASA (Sigma, 800 mg/ml), IBR (Sigma, 800 mg/ml) and DIC (Sigma, 800 mg/ml) were freshly prepared in dimethyl sulfoxide (DMSO, Sigma). In drug combination experiments of planktonic cells, 0.031 to 8 mg/ml for ASA, IBR and DIC, 0.0156 to 64 μg/ml for FLU, 0.002 to 8 μg/ml for ITC and AMB, 0.0002 to 1 μg/ml for VOR and 0.008 to 32 μg/ml for CAS were used. For biofilms experiments, FLU, ITC, VOR and AMB were all used at a final concentration ranging from 2 to 1024 μg/ml. CAS was used at a final concentration ranging from 0.016 to 64 μg/ml. ASA, IBR and DIC was used at a final concentration ranging from 0.031 to 8 mg/ml. Both in planktonic form and biofilm study, the DMSO final concentrations in each cell were below 1%.

### Measurement of antifungal susceptibility testing of planktonic cells

The MICs of the NSAIDs and antifungal drugs were tested using the broth microdilution method in 96-well plates (Corningμ, NY 14831, USA,) according to the Clinical and Laboratory Standards Institute (CLSI) standard M27-A3, and the MICs of azoles and NSAIDs were expressed as the minimal concentration in the well showing ≤ 50% growth for planktonic cells compared to positive control wells and complete inhibition endpoints for AMB (100%). Negative controls were performed with only RPMI 1640 in each well, and positive controls were performed with only micro-organisms in the wells. Interactions between the NSAIDs and antifungals were studied using the checkerboard microdilution technique in 96-well microtitration plates. Each isolate was tested three times on different days. Either alone or in combination tests for planktonic cells, MICs were determined visually following M27-A3 after incubation at 35°C for 48 h.

### Biofilm development and susceptibility testing

100 μl of adjusted *T*. *asahii* suspension (10^6^ CFU/ml) was transferred into each well of the 96-well microtiter plates, followed by an adhesion phase at 37°C for 1 h. The wells containing RPMI 1640-MOPS medium without *T*. *asahii* served as background controls. After the adhesion phase, each well was washed twice lightly with sterile PBS to remove non-adherent cells and then refilled with 200 μl of fresh prepared RPMI 1640-MOPS medium for 24 h of incubation at 37°C. Subsequently, the biofilms were washed twice with PBS in preparation for adding drugs. The effects of NSAIDs and antifungals in combination were assessed using the broth microdilution checkerboard technique [16,17]. After incubating these plates for 48 h at 37°C, the prewashed biofilms and background control wells were quantified using a 2, 3-bis (2-methoxy-4-nitro-5-sulfophenyl)-5-[(phenylamino) carbonyl]-2H-tetra-zolium hydroxide (XTT) (Sigma) reduction assay [18]. From the colorimetric readings and after subtracting the corresponding values for background controls, the sessile minimum inhibitory concentrations (SMICs) were determined for each fungal isolate, which are the antifungal concentrations at which there is a ≥ 50% decrease in absorbance, compared with the control biofilms (wells in the absence of antifungal drug) [17]. All of the assays were repeated three times on separate occasions.

### Drug interaction modeling

The data obtained using the spectrophotometric method were analyzed using two models: the Loewe additivity (LA) and the Bliss independence (BI) methods [19]. The FICI model is based on the LA model, as follows. The fractional inhibitory concentration index (FICI) was calculated using the equation FICI = (A_c_/ A_a_) + (B_c_/ B_a_), where A_c_ and B_c_ are the MICs of drugs A and B in combination, respectively, and A_a_ and B_a_ are the MICs of the drugs alone, respectively. FICIs of ≤ 0.5 indicate synergy, FICIs of > 4 indicate antagonism, and FICIs of >0.5 and ≤ 4 indicate no interaction.

The BI theory is based on the idea that two drugs act independently, in the probabilistic sense of independence [19]. Referring to previous research [20], *ΔE* was calculated as follows: *I*_*A*_ or *I*_*B*_ = the experimental percentages of inhibition of each drug acting alone *I*_i_ = (*I*_A_+*I*_B_)-(*I*_A_×*I*_B_) and *I* = 1-*E*; thus, the following equation is derived: *E*_i_ (predicted percentage of growth) = *E*_A_×*E*_B_, where *E*_A_ or *E*_B_ = the experimental percentages of growth of each drug acting alone. *E*_measured_ = the experimental percentages of growth of each drug combination. *ΔE* = *E*_i_-*E*_measured_. A three-dimensional plot with the *ΔE* depicted on the z axis results in a surface plot (see S1 Fig). Each combination was tested three times on different days. When the average *ΔE* of the three replicates with each separate interaction was positive or negative, and its 95% confidence interval (CI) did not include 0, synergy or antagonism was claimed. In any other case, indifference was concluded.

## Results

### Inhibitory effect of the NSAIDs on the planktonic and biofilm cells of *T*. *asahii*

Weak inhibitions were found when the NSAIDs were used alone, for both planktonic and biofilm cells of *T*. *asahii*. The MICs ranges of ASA, IBR and DIC were ≥ 8 mg/ml, 0.5–2 mg/ml and 1–4 mg/ml, respectively (Table 1). The SMICs of ASA, IBR and DIC were >8 mg/ml, 1–4 mg/ml and >8 mg/ml (Table 2), respectively, which indicated the decreased susceptibility of the biofilm cells.

**Table 1:** Susceptibilities of planktonic cells of the 15 *Trichosporon asahii* isolates against NSAIDs alone and in combination with antifungal agents and the percentage of interpretation effects of each combination (n = 3).

Drug combination	MIC(range) of drug				FICI		Interpretation		
	Alone		In combination		Median	Range	Syn	Ind	Ant
	NSAID(mg/ml)	Antifungal(μg/ml)	NSAID(mg/ml)	Antifungal(μg/ml)					
ASA/FLU	8–16	4–32	0.125–2	0.063–16	0.516	0.063–1.250	7(46.67%)	8(53.33%)	0(0.00%)
ASA/ITC	8–16	0.125–1	0.125–4	0.015–0.5	0.508	0.136–1.008	6(40.00%)	9(60.00%)	0(0.00%)
ASA/VOR	8–16	0.031–0.125	0.125–8	0.004–0.125	0.750	0.250–1.125	4(26.67%)	11(73.33%)	0(0.00%)
ASA/AMB	8–16	0.5–4	0.125–4	0.031–2	0.313	0.141–0.625	12(80.00%)	3(20.00%)	0(0.00%)
ASA/CAS	8–16	8–32	0.25–2	2–16	0.375	0.156–1.125	9(60.00%)	6(40.00%)	0(0.00%)
IBR/FLU	0.5–2	4–32	0.031–1	0.125–8	0.375	0.141–1.250	10(66.67%)	5(33.33%)	0(0.00%)
IBR/ITC	0.5–2	0.125–1	0.125–0.5	0.031–0.5	0.500	0.188–1.250	8(53.33%)	7(46.67%)	0(0.00%)
IBR/VOR	0.5–2	0.031–0.125	0.063–1	0.001–0.063	0.750	0.313–1.250	4(26.67%)	11(73.33%)	0(0.00%)
IBR/AMB	0.5–2	0.5–4	0.031–0.5	0.063–1	0.250	0.063–0.750	13(86.67%)	2(13.33%)	0(0.00%)
IBR/CAS	0.5–2	8–32	0.016–0.125	1–8	0.188	0.063–1.016	11(73.33%)	4(26.67%)	0(0.00%)
DIC/FLU	1–4	4–32	0.031–0.5	0.5–16	0.516	0.070–1.250	6(40.00%)	9(60.00%)	0(0.00%)
DIC/ITC	1–4	0.125–1	0.031–2	0.031–0.5	0.750	0.156–2.000	4(26.67%)	11(73.33%)	0(0.00%)
DIC/VOR	1–4	0.031–0.125	0.063–2	0.001–0.125	0.750	0.500–1.500	2(13.33%)	13(86.67%)	0(0.00%)
DIC/AMB	1–4	0.5–4	0.031–1	0.125–1	0.313	0.094–0.750	12(80.00%)	3(20.00%)	0(0.00%)
DIC/CAS	1–4	8–32	0.125–2	4–8	0.750	0.25–1.25	5(33.33%)	10(66.67%)	0(0.00%)

MIC, the concentration causing a 50% reduction in optical density of the planktonic cells compared with the optical density of the untreated cells using the same isolates; FICI, fractional inhibitory concentration index, FICI≤0.5, synergy; FICI>0.5–4, indifference; FICI>4, antagonism; Median, the median of the FICIs with one type of combination; Range, the range of the FICIs with one type of combination; Syn, a combination indicating synergistic interaction; Ind, a combination indicating indifferent interaction; Ant, a combination indicating antagonistic interaction; FLU, Fluconazole; ITC, Itraconazole; VOR, Voriconazole, AMB, amphotericin B; CAS, caspofungin; ASA, aspirin; IBR, ibuprofen; DIC, diclofenac sodium

**Table 2:** Susceptibilities of biofilm cells of the 15 *Trichosporon asahii* isolates against NSAIDs alone and in combination with antifungal agents and the percentage of interpretation effects of each combination (n = 3).

Drug combination	SMIC(range) of drug				FICI		Interpretation		
	Alone		In combination		Median	Range	Syn	Ind	Ant
	NSAID(mg/ml)	Antifungal(μg/ml)	NSAID(mg/ml)	Antifungal(μg/ml)					
ASA/FLU	16	512–1024	0.5–8	64–512	0.375	0.094–1	8(53.33%)	7(46.67%)	0(0.00%)
ASA/ITC	16	1024	0.5–8	128–1024	0.563	0.188–1.031	5(33.33%)	10(66.67%)	0(0.00%)
ASA/VOR	16	1024	0.5–8	256–1024	0.625	0.281–1.031	2(13.33%)	13(86.67%)	0(0.00%)
ASA/AMB	16	128–1024	0.5–8	32–128	0.281	0.063–0.625	10(66.67%)	5(33.33%)	0(0.00%)
ASA/CAS	16	16–64	4–8	8–16	0.500	0.375–1.5	8(53.33%)	7(46.67%)	0(0.00%)
IBR/FLU	2–4	512–1024	0.25–1.0	32–1024	0.375	0.094–1.125	9(60.00%)	6(40.00%)	0(0.00%)
IBR/ITC	2–4	1024	0.5–4	4–1024	0.625	0.156–1.5	6(40.00%)	9(60.00%)	0(0.00%)
IBR/VOR	2–4	1024	0.5–2	32–1024	0.750	0.281–1.5	3(20.00%)	12(80.00%)	0(0.00%)
IBR/AMB	2–4	128–1024	0.03–2	16–256	0.188	0.047–0.750	11(73.33%)	4(26.67%)	0(0.00%)
IBR/CAS	2–4	16–64	0.25–2	2–8	0.375	0.094–1.063	9(60.00%)	6(40.00%)	0(0.00%)
DIC/FLU	16	512–1024	0.5–8	16–1024	0.500	0.141–2	9(60.00%)	6(40.00%)	0(0.00%)
DIC/ITC	16	1024	1.0–8.0	128–1024	0.625	0.25–2	7(46.67%)	8(53.33%)	0(0.00%)
DIC/VOR	16	1024	1.0–4.0	32–1024	0.531	0.25–1.25	7(46.67%)	8(53.33%)	0(0.00%)
DIC/AMB	16	128–1024	0.125–8	4.0–256	0.313	0.094–1.25	10(66.67%)	5(33.33%)	0(0.00%)
DIC/CAS	16	16–64	1–4	4–16	0.5	0.375–1.5	8(53.33%)	7(46.67%)	0(0.00%)

SMIC, the sessile MIC, the concentration causing a 50% reduction in XTT reduction assay of the biofilm cells compared with the untreated cells using the same isolates; FICI, fractional inhibitory concentration index, FICI≤0.5, synergy; FICI>0.5–4, indifference; FICI>4, antagonism; Median, the median of the FICIs with one type of combination; Range, the range of the FICIs with one type of combination; Syn, a combination indicating synergistic interaction; Ind, a combination indicating indifferent interaction; Ant, a combination indicating antagonistic interaction; FLU, Fluconazole; ITC, Itraconazole; VOR, Voriconazole, AMB, amphotericin B; CAS, caspofungin; ASA, aspirin; IBR, ibuprofen; DIC, diclofenac sodium

### Interactive effects of drug combinations on the planktonic and biofilm cells of *T*. *asahii*

In the planktonic form of the fifteen isolates, the lowest FICI values were found in IBR/ AMB (range, 0.063–0.75; median, 0.25) and IBR/ CAS (range, 0.063–1.016; median, 0.188) in all combinations (see Table 1). The MICs of AMB decreased from 0.5–4 μg/ml to 0.063–1 μg/ml with IBR and the MICs of CAS decreased from 8–32 μg/ml to 1–8 μg/ml with IBR. Conversely, the SMICs of AMB reduced from 128–1024 μg/ml to 32–128 μg/ml with ASA and to 16–256 μg/ml with IBR, which showed the lowest FICI values in ASA/ AMB (range, 0.063–0.625; median, 0.281) and IBR/ AMB (range, 0.047–0.750; median, 0.188) in Table 2. None of the data sets analyzed had FICIs higher than 2, indicating that antagonism was not observed.

NSAIDs / FLU had high synergistic interaction percentages for six HFM-isolates *T*. *asahii* isolates: 83.33% for ASA, 100% for IBR and 83.33% for DIC (see Table 3). The MICs of NSAID with either ITC (0.25–4 mg/ml for ASA, 0.125–0.25 mg/ml for IBR, 0.06–1 mg/ml for DIC) or VOR (0.25–8 mg/ml for ASA, 0.06–0.5 mg/ml for IBR, 0.06–2 mg/ml for DIC) were not lower than NSAID/FLU (0.13–2 mg/ml for ASA, 0.03–0.5 mg/ml for IBR, 0.03–0.5 mg/ml for DIC).

**Table 3:** The susceptibility to antifungals alone and the combination of FLU/ NSAIDs on CBS 2479 and 6 Flu-resistant isolates.

isolates	NSAIDs	FLU	ITC	VOR	AMB	NSAIDs/	FLU	FICI	NSAIDs/	ITC	FICI	NSAIDs/	VOR	FICI	NSAIDs/	AMB	FICI
ASA+antifungals																	
2479	8.00	4.00	0.25	0.03	1.00	2.00	1.00	0.500	1.00	0.13	0.625	2.00	0.02	0.750	2.00	0.13	0.375
n1	8.00	16.00	1.00	0.13	1.00	1.00	2.00	0.250	4.00	0.25	0.750	0.50	0.03	0.313	0.50	0.13	0.188
n2	8.00	32.00	0.50	0.13	1.00	0.13	2.00	0.078	1.00	0.25	0.625	0.25	0.06	0.531	0.25	0.13	0.156
n3	8.00	32.00	1.00	0.06	0.50	2.00	8.00	0.500	2.00	0.50	0.750	4.00	0.02	0.740	0.25	0.13	0.281
n4	8.00	32.00	1.00	0.13	1.00	0.25	1.00	0.063	0.25	0.13	0.156	8.00	0.00	1.031	0.13	0.13	0.141
n5	8.00	16.00	0.13	0.13	1.00	1.00	16.00	1.125	2.00	0.06	0.750	1.00	0.03	0.375	2.00	0.13	0.361
n6	8.00	32.00	0.25	0.13	4.00	0.13	2.00	0.078	4.00	0.13	1.000	1.00	0.02	0.250	0.25	2.00	0.531
IBR+antifungals																	
2479	1.00	4.00	0.25	0.03	1.00	0.25	0.25	0.313	0.25	0.06	0.500	0.25	0.03	1.250	0.13	0.13	0.250
n1	1.00	16.00	1.00	0.13	1.00	0.25	0.13	0.258	0.25	0.50	0.750	0.25	0.01	0.314	0.13	0.13	0.250
n2	1.00	32.00	0.50	0.13	1.00	0.03	8.00	0.281	0.25	0.50	1.250	0.50	0.03	0.750	0.03	0.13	0.156
n3	1.00	32.00	1.00	0.06	0.50	0.03	8.00	0.281	0.125	0.25	0.375	0.06	0.02	0.313	0.03	0.06	0.156
n4	1.00	32.00	1.00	0.13	1.00	0.25	1.00	0.281	0.25	0.25	0.500	0.50	0.06	1.000	0.25	0.25	0.500
n5	1.00	16.00	0.13	0.13	1.00	0.25	2.00	0.375	0.25	0.03	0.500	0.25	0.01	0.314	0.50	0.25	0.750
n6	2.00	32.00	0.25	0.13	4.00	0.50	1.00	0.281	0.125	0.03	0.188	0.25	0.03	0.375	0.13	0.50	0.188
DIC+antifungals																	
2479	2.00	4.00	0.25	0.03	1.00	0.50	4.00	1.250	0.50	0.25	1.250	2.00	0.00	1.032	0.13	0.25	0.313
n1	4.00	16.00	1.00	0.13	1.00	0.03	1.00	0.070	0.50	0.06	0.188	0.25	0.13	1.063	0.50	0.25	0.375
n2	2.00	32.00	0.50	0.13	1.00	0.13	0.50	0.078	0.50	0.25	0.750	0.06	0.06	0.531	1.00	0.19	0.688
n3	2.00	32.00	1.00	0.06	0.50	0.50	8.00	0.500	1.00	0.50	1.000	2.00	0.01	1.128	0.25	0.25	0.625
n4	2.00	32.00	1.00	0.13	1.00	0.03	8.00	0.266	0.25	0.25	0.375	0.50	0.03	0.500	0.03	0.13	0.141
n5	4.00	16.00	0.13	0.13	1.00	0.03	8.00	0.508	0.25	0.03	0.313	1.00	0.06	0.750	0.50	0.13	0.250
n6	2.00	32.00	0.25	0.13	4.00	0.03	8.00	0.266	0.06	0.03	0.156	0.25	0.06	0.625	0.50	0.50	0.375

FLU, Fluconazole; ITC, Itraconazole; VOR, Voriconazole; AMB, amphotericin B; ASA, aspirin; IBR, Ibuprofen; DIC, diclofenac sodium

The results of the checkerboard analysis interpreted using the nonparametric methods based on the LA theory (FICI) are summarized in Table 1 (planktonic cells) and Table 2 (biofilms). In the checkerboard microtiter plate format, the highest percentage of synergistic interactions was 86.67% for IBR / AMB against the planktonic forms and 73.33% for IBR / AMB against the biofilms (see Table 1 and Table 2).

### Comparison of LA and BI theories

There was good agreement between the FICI and BI models for the antifungal ⁄ NSAID interactions (Table 4). Compared with FICI, the rates according to BI theory reached 86.67% for the fifteen combinations against CBS 2479. DIC / ITC indicated indifferent interactions according to FICI, whereas the average *ΔE* was positive and its 95% CI among the three replicates did not include 0 and had a median ΣSyn >100%, which revealed a moderate synergistic action according to the BI model [19]. The synergistic interaction, which was observed from DIC / VOR using the FICI model, showed indifference using the BI method.

**Table 4:** Comparison of the LA and BI model on all combinations against biofilm cell of *T*. *asahii* CBS2479.

drug combinations	LA model (FICI)			BI model (*ΔE*)		
	Median	Range	INT	Average	95% CI	INT
ASA/FLU	0.5	0.266–0.515	SYN	10.01	5.19–14.83	SYN
ASA/ITC	0.625	0.075–1.031	IND	3.33	-0.64–7.31	IND
ASA/VOR	0.75	0.5–1.5	IND	1.54	-0.33–3.41	IND
ASA/AMB	0.375	0.188–0.5	SYN	21.09	5.69–36.50	SYN
ASA/CAS	1	0.75–1	IND	1.32	-0.34–2.98	IND
IBR/FLU	0.5	0.375–0.5	SYN	4.54	2.55–6.53	SYN
IBR/ITC	0.156	0.141–0.531	SYN	0.75	0.32–1.17	SYN
IBR/VOR	0.281	0.156–0.313	SYN	8.47	7.48–9.47	SYN
IBR/AMB	0.375	0.125–0.75	SYN	17.01	7.36–26.67	SYN
IBR/CAS	0.375	0.25–0.375	SYN	2.53	1.60–3.45	SYN
DIC/FLU	0.25	0.141–0.75	SYN	16.25	5.92–26.59	SYN
DIC/ITC	0.625	0.25–0.75	IND	7.08	4.86–9.29	SYN
DIC/VOR	0.313	0.094–0.625	SYN	1.01	-0.15–2.16	IND
DIC/AMB	0.375	0.281–0.375	SYN	12.34	6.03–18.65	SYN
DIC/CAS	0.75	0.625–0.75	IND	0.21	-0.05–0.47	IND

LA and BI were two drug interaction models. FICI values are shown as the median of three independent experiments. For the ΔE model. Each combination was tested three times on different days. INT, interaction; FLU, fluconazole; ITC, itraconazole; VOR, voriconazole; AMB, amphotericin B; CAS, caspofungin; ASA, aspirin; IBR, ibuprofen; DIC, diclofenac sodium; SYN, synergism; ANT, antagonism; IND, indifference.

## Discussion

According to previous reports, even when antifungal drugs that are administered, the mortality from invasive trichosporonosis is high (from 42% to 82%) [3,10,21–23]. Pan-resistance of *Trichosporon spp*. has been reported with high MICs of antifungals (flucytosine > 64 μg/ml, FLU > 256 μg/ml, ITC > 16 μg/ml, VOR > 16 μg/ml, posaconazole > 8 μg/ml, CAS > 16 μg/ml), which may seriously threaten the health of immunocompromised patients [7]. Susceptibility testing results showed that all 9 clinical isolates exhibited diverse MICs of azoles. FLU was less active than ITC and VOR, with a range of 4 to 8 μg/ml (Table 1). VOR was the most effective agent against planktonic *T*. *asahii* (0.03 to 0.12 μg/ml) *in vitro*, as reported previously for *T*. *asahii* isolates [24]. It was also observed that when used alone, the highest MIC of AMB was 8 μg/ml (Table 1), according to a previous study with high MICs of amphotericin B of *T*. *asahii* (≥ 2 μg/ml) [5]. In previous study, against *C*. *neoformans* (MIC, 16–64 μg/ml) and *T*. *asahii* (MIC, 8–32 μg/ml, Table 1) CAS was inactive [25]. The above experimental results suggest the necessity of the drug combination therapies.

As for our study on planktonic cells, the NSAIDs alone can inhibit *T*. *asahii* at 8 mg/ml for ASA, 0.5 mg/ml for IBR and 1 mg/ml for DIC. A previous study observed that the MICs of sodium salicylate and DIC of *C*. *albicans* were > 0.256 mg/ml [26]. Sebolai et al observed that at 0.54 mg/ml ASA, the growth of all *Cryptococcus* species was significantly inhibited [13], and similar effects were found for *Eremothecium spp*. and other yeasts [27]. In combinations, the NSAIDs can obviously lower the MICs of the five antifungals for *T*. *asahii*. When the antifungal agents were combined with NSAIDs, especially for AMB, a noteworthy synergistic effect was revealed both in planktonic and biofilm cells. Regarding the planktonic cells of the 15 isolates, the AMB / IBR combination showed the best synergistic effect, with the MICs of AMB and IBR decreasing from 0.5–4 μg/ml to 0.063–1 μg/ml and from 0.5–2 mg/ml to 0.031–0.5 mg/ml respectively. Analogously, in the presence of AMB / IBR, the SMICs of each drug were reduced from 128–1024 μg/ml to 16–256 μg/ml and 2–4 mg/ml to 0.03–2 mg/ml respectively, which also indicated the best synergistic effect. The combination of AMB / IBR (86.67% for planktonic and 73.33% for biofilms) yielded the highest percentages of synergistic interactions; in contrast, the lowest synergistic effects were observed for DIC / VOR (13.33% for planktonic) and ASA / VOR (13.33% for biofilms). It is worth mentioning that the 5 isolates were 100% inhibited when applied in safe plasma drug concentrations in the AMB / IBR combination (Table 1). When analyzed using FICI and the *ΔE* model, synergistic effects were also found for the ASA / AMB combination against biofilms of *C*. *albicans* [20].

Most of the MICs of NSAIDs in the present study, either alone or combined with antifungals except CAS (Table 1), were still greater in number than the highest plasma drug concentrations safely used in clinical practice; however, our study can still provide some guidance for future research on clinical combination therapies with NSAIDs / antifungals. Some drug combinations in our study showed obvious inhibitions of some *T*. *asahii* isolates when the concentration was under the safe plasma drug concentrations (e.g., IBR 0.031 mg/ml when combined with AMB, see Table 1), which means that the physiological concentrations of IBR/ AMB can inhibit those clinical isolates. Second, derivatives of NSAIDs, which were able to achieve MICs under safe plasma drug concentrations, may be developed in the future. A previous study found that derivatives of IBR and biphenyl-4-yloxy acetic acid showed moderate antimicrobial activity against tested bacterial and fungal [28]. All of the tested compounds showed diverse antifungal activities against *C*. *albicans* (MIC values of 12.5–200 μg/mL) compared to the antifungal activities of the antifungal drug ketoconazole against *C*. *albicans* (MIC, 6.25 μg/mL). Thus, the derivatives of NSAIDs may have important prospect in later antifungal research, which also substantiates our claim [28]. In addition, Pina-Vaz et al. implied that the concentration of ibuprofen (0.1 mg/ml) was also above the safe plasma drug concentration when combined with fluconazole to reach 50% inhibition against *C*. *albicans* [29,30]; however, their recent study found that the combination with FLU and IBR in safe plasma drug concentrations (IBR with 10 mg/kg of body weight/ day, FLU with 8 mg/kg / day) resulted in the clearance of *C*. *albicans* systemic infections and reversed *in vivo* FLU resistance, which indicated a clinically practical possibility for IBR [31]. Referring to these experiments, we can speculate that IBR or other NSAIDs may have the same *in vivo* potent synergic effect with antifungals with lower concentrations than when they revert to antifungal resistance of *T*. *asahii in vitro*.

Echinocandins act by inhibiting the biosynthesis of β-1, 3 glucan, which leads to disruption of the fungal cell wall [32]. The synergistic interactions percentage of CAS / NSAIDs in our research was 33.33%–73.33% for the planktonic form. The mechanism of synergy caused by CAS / NSAIDs may be a result of CAS-mediated weakening of the cell wall that leads to facilitated NSAIDs-targeting of fungal membranes [33], which in turn reinforces the antifungal activity of CAS.

It is undeniable that some isolates with a high FLU MIC value may also be a threat to clinical treatment against *T*. *asahii* infections [8,34]. In our research, NSAIDs showed high percentages of synergistic effects with FLU against planktonic cells of six *T*. *asahii* HFM-isolates. In contrast, diverse MICs of antifungals and FICIs for the six induced HFM-isolates were characterized (see Table 4). Several mechanisms of azole resistance in *C*. *albicans*, including reduced accumulation of the drugs through over expression of CDR1,CDR2 and MDR1, alteration or over-expression of the target enzyme (14α-sterol-demethylase, ERG11) and loss of function downstream mutation in the ergosterol pathway (defective Δ-5,6-desaturase encoded by ERG3), have already been described [35]. A previous drug combination study found that it was possible that IBR and sodium salicylate (SS) had an effect on *C*. *albicans* cell membranes by facilitating the uptake of the azoles to enhance the efficiency of the azoles [36]. When the blockade of the pumps was achieved using IBR (0.1 mg/ml), a concomitant revision of resistance was registered, with a decrease in the MIC values of FLU from 128–256 μg/mL to 1–128 μg/mL (i.e., a decrease of 2–128 times) and VOR from 4–16 μg/mL to 0.015–4 μg/mL (i.e., a decrease of 8–512 times) for *Candida* species [30]. Compared to *C*. *albicans*, only one study on an antifungal-resistant mechanism of *T*. *asahii* reported that the antifungal-resistant mechanism may be associated with an ERG11 mutation [15].

As first-line drugs in the treatment of invasive trichosporonosis infections [37], azoles had been observed in relation to the resistant phenomenon of *T*. *asahii* in recent years [7,8,34]. In previous studies, the biofilms formed by *T*. *asahii* were resistant to all of the azoles tested (MIC > 1024 μg/ml) and were up to 16,000 times more resistant to VOR than planktonic cells [9]. Meanwhile, biofilm-associated invasive trichosporonosis may lead to persistent or recurrent infections with high mortality despite antifungal treatment [1,9]. Consistent with the above results, our study also found that biofilm cells of *T*. *asahii* were intrinsically resistant to the tested antifungal agents (FLU, ITC, VOR and AMB) (Table 2), which might lead to the failure of clinical treatment. No appropriate SMIC of antifungal agents except CAS, when combined with the three NSAIDs, was observed for clinical applications. Multiple factors contribute to the elevated drug resistance of pathogenic fungal biofilms, including the increased expression of drug efflux pumps, increased cell density, and elevated β-1,3 glucan levels in the cell wall and biofilm matrix, as well as signaling mediated by protein kinase C and the protein phosphatase calcineurin [38,39]. For *T*. *asahii* biofilms, it was indicated that EPS was associated with the lack of activity of antifungals observed for *T*. *asahii* biofilms in that model [9].

In the present study, we found that NSAIDs were able to inhibit the growth of *T*. *asahii* biofilm cells. Other studies have observed that significant inhibitive effects of ASA on growth and biofilm formation of *Candida spp*. were achieved only with suprapharmacological concentrations of ASA (0.39–1.56 mg/ml)[40]. It has been reported that because ASA and other NSAIDs are COX inhibitors, they were able to reduce the growth of planktonic and biofilm cells of *C*. *albicans* with ASA showing the greatest effects, which was related to decreased prostaglandin levels [41]. ASA has been reported to suppress the morphogenesis of *C*. *albicans* hyphae and filamentous structures, which are critical structures of biofilms [42], by inhibiting the production of 3(R)-hydroxyoxylipins, oxygenated fatty acid metabolites derived from arachidonic acid [43]. Because β-1, 3 glucan is essential for the extracellular matrix of fungal biofilms [42], CAS was also shown to be active against biofilms of *T*. *asahii* in the present study, with SMICs of 16–64 μg/ml compared to azoles (SMICs ≥ 512 μg/ml) and AMB (SMICs ≥ 128 μg/ml). The SMICs of CAS reduced from 16–64 μg/ml to 2–8 μg/ml with 0.25–2 mg/ml IBR. Anna Bink et.al observed that 0.6 mg/ml DIC reduced the SMIC of CAS from 6.56 μg/ml to 0.82 μg/ml on pretreated biofilms of *C*. *albicans* CAF2-1, which suggested that DIC and other NSAIDs may induce an increase in the membrane permeability of the *C*. *albicans* biofilm and thus enhance the inhibition of CAS [33,44].

Using *in vitro* and *in vivo* interaction studies, FLU, ITC and VOR were found to have obvious inhibitions on the cytochrome P450 (CYP) enzyme system and may hinder the human drug metabolism of NSAIDs (including ASA, IBR and DIC) [45–47]. VOR and FLU treatments have increased the level of exposure to S-(+)-IBR 2- and 1.8-fold, respectively [46]. Nevertheless, the echinocandins have shown no marked inhibition of P450 activities, except for some inhibition of CYP3A4/5 activity [47]. The blood/plasma concentrations of concomitant drugs were not markedly affected by coadministration of echinocandins *in vivo* [47], suggesting that echinocandins do not cause clinically significant interactions with drugs that are metabolized by P450s via the inhibition of metabolism. To our knowledge, there are no published articles that affirm that AMB inhibits the CYP enzyme system. The differential effects of these antifungal agents on P450 activities must be considered when clinicians select antifungal agents for patients who are also receiving NSAIDs.

Another point is that when we combined the antifungals with the NSAIDs against *T*. *asahii* biofilms in our study, the SMICs of the azoles alone were occasionally above the maximum concentration, according to the checkerboard method (Table 2), which has presented problems when calculating the precise FICI values. However, the *ΔE* model can help yield results. Good agreement was found with the FICI model (the consistent rate was 86.67%). Thus, the *ΔE* model should be considered an option when the FICI cannot be calculated precisely in *T*. *asahii* biofilms research.

## Conclusions

The present study found that the fifteen types of combinations studied yielded diverse percentages of synergistic interactions (13.33%–86.67% for planktonic cells, 13.33%–73.33% for biofilm cells), and no antagonistic interaction was observed. The *ΔE* model may be a more efficient method to analyze the drug combination effects on biofilm cells. Our data indicate that the use of NSAIDs in combination with antifungals, especially IBR/ AMB and IBR/ CAS, may be a therapy strategy to treat invasive *Trichosporon* infections. Further *in vivo* / animal study and relevant mechanisms need to be investigated.

## Supporting Information

S1 Fig Assessment of *in vitro* interaction between ibuprofen (IBR) and amphotericin B (AMB) against *Trichosporon asahii* (CBS 2479) biofilm using the LA-based model and the BI-based model. (A) Checkerboard showing the percentage of biofilm growth for each combination using XTT reduction assay. (B) Three-dimensional (1) and contour (2) plots of the percent synergy calculated with the nonparametric approach.
(DOCX)
