# Supplementary material for: In Vitro Interactions between Non-Steroidal Anti-Inflammatory Drugs and Antifungal Agents against Planktonic and Biofilm Forms of Trichosporon asahii
Source: PLoS One. 2016 Jun 8;11(6):e0157047. doi: 10.1371/journal.pone.0157047 (PMC4898695; doi:10.1371/journal.pone.0157047)
Supplement: S1 Fig — (A) Checkerboard showing the percentage of biofilm growth for each combination using XTT reduction assay. (B) Three-dimensional (1) and contour (2) plots of the percent synergy calculated with the nonparametric approach. (DOCX) [file pone.0157047.s001.docx]

**S1 Fig. Assessment of *in vitro* interaction between ibuprofen (IBR) and amphotericin B (AMB) against *Trichosporon asahii* (CBS 2479) biofilm using the LA-based model and the BI-based model.**


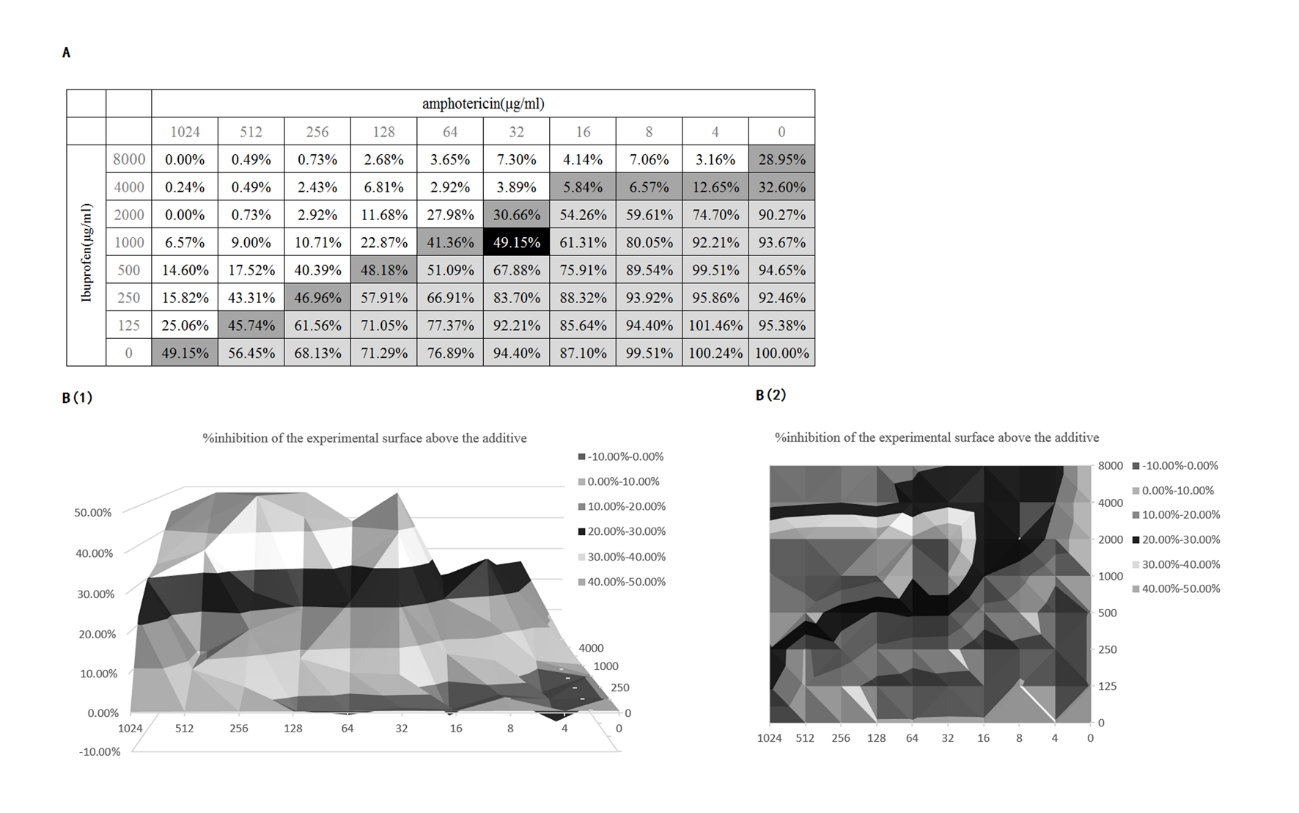


(A) Checkerboard showing the percentage of biofilm growth for each combination using XTT reduction assay. The iso-effective combinations based on which the ∑FIC indices were calculated for the lowest ∑FIC index (the black cell). (B) Three-dimensional (1) and contour (2) plots of the percent synergy calculated with the nonparametric approach. The difference between the predicted and measured percentages of fungal growth (Δ*E* =*E*_predicted_ - *E*_measured_) is shown on the z axis.
